# Identification and characterization of wheat long non-protein coding RNAs responsive to powdery mildew infection and heat stress by using microarray analysis and SBS sequencing

**DOI:** 10.1186/1471-2229-11-61

**Published:** 2011-04-07

**Authors:** Mingming Xin, Yu Wang, Yingyin Yao, Na Song, Zhaorong Hu, Dandan Qin, Chaojie Xie, Huiru Peng, Zhongfu Ni, Qixin Sun

**Affiliations:** 1 State Key Laboratory for Agrobiotechnology and Key Laboratory of Crop Heterosis and Utilization (MOE) and Key Laboratory of Crop Genomics and Genetic Improvement (MOA), Beijing Key Laboratory of Crop Genetic Improvement, China Agricultural University, Beijing, 100094, PR China; 2 National Plant Gene Research Centre (Beijing), Beijing 100094, PR China; 3 Department of Plant Genetics & Breeding, China Agricultural University, Yuanmingyuan Xi Road No. 2, Haidian District, Beijing, 100193, PR China

## Abstract

**Background:**

Biotic and abiotic stresses, such as powdery mildew infection and high temperature, are important limiting factors for yield and grain quality in wheat production. Emerging evidences suggest that long non-protein coding RNAs (npcRNAs) are developmentally regulated and play roles in development and stress responses of plants. However, identification of long npcRNAs is limited to a few plant species, such as Arabidopsis, rice and maize, no systematic identification of long npcRNAs and their responses to abiotic and biotic stresses is reported in wheat.

**Results:**

In this study, by using computational analysis and experimental approach we identified 125 putative wheat stress responsive long npcRNAs, which are not conserved among plant species. Among them, some were precursors of small RNAs such as microRNAs and siRNAs, two long npcRNAs were identified as signal recognition particle (SRP) 7S RNA variants, and three were characterized as U3 snoRNAs. We found that wheat long npcRNAs showed tissue dependent expression patterns and were responsive to powdery mildew infection and heat stress.

**Conclusion:**

Our results indicated that diverse sets of wheat long npcRNAs were responsive to powdery mildew infection and heat stress, and could function in wheat responses to both biotic and abiotic stresses, which provided a starting point to understand their functions and regulatory mechanisms in the future.

## Background

The developmental and physiological complexity of eukaryotes could not be explained solely by the number of protein-coding genes [1]. For example, the *Drosophila melanogaster *genome contains only twice as many genes as some bacterial species, although the former is far more complex in its genome organization than the latter. Similarly, the number of protein-coding genes in human and nematode is extremely close. A portion of this paradox can be resolved through alternative pre-mRNA splicing [2]. In addition, post-translational modifications can also contribute to the increased complexity and diversity of protein species [3].

Recent studies suggest that most of the genome are transcribed, among the transcripts only a small portion encode for proteins, whereas a large portion of the transcripts do not encode any proteins, which are generally termed non-protein coding RNAs (npcRNA). For example, transcriptome profiling in rice (*Oryza sativa*) indicates that there are about 8400 putative npcRNAs, which do not overlap with any predicted open reading frames (ORFs) [4]. These npcRNAs are subdivided as housekeeping npcRNAs (such as transfer and ribosomal RNAs) and regulatory npcRNAs or riboregulators, with the latter being further divided into short regulatory npcRNAs (<300 bp in length, such as microRNA, siRNA, piwi-RNA) and long regulatory npcRNAs (>300 bp in length). With the identification of microRNAs and siRNAs in diverse organisms, increasing evidences indicate that these short npcRNAs play important roles in development, responses to biotic and abiotic stresses by cleavage of target mRNAs or by interfering with translation of target genes [5-9].

Long npcRNAs are transcribed by RNA polymerase II, polyadenylated and often spliced [10]. Studies in mice and human suggested that at least 13% and 26% of the unique full-length cDNAs, respectively, are thought to be poly(A) tail-containing long npcRNAs [11-13]. Emerging evidences also suggest that long npcRNAs are developmentally regulated and responsive to external stimuli, and play roles in development and stress responses of plants and disease in human. For example, some long npcRNAs are regulated in various stresses in plants and animals [9,14-16]. In *Caenorhabditis elegans*, 25 npcRNAs are either over- or under-expressed under heat shock or starvation conditions [17], while in *Arabidopsis*, the abundance of 22 putative long npcRNAs are regulated by phosphate starvation, salt stress or water stress [18]. In *Arabidopsis*, long npcRNA, *COOLAIR *(cold induced long antisense intragenic RNA), is cold-induced FLC antisense transcripts, and has an early role in the epigenetic silencing of FLC and to silence FLC transcription transiently [19]. Long npcRNA *HOTAIR *in human is reported to reprogram chromatin state to promote cancer metastasis [20].

Currently, two computational methods are employed to identify long npcRNAs, genome-based and transcript-based. Using genomic sequences, more than 200 candidate long npcRNAs were predicted in *Escherichia coli *[21], and at least 20 long npcRNA genes have been experimentally confirmed [22]. In *Rhizobium etli*, 89 candidate npcRNAs are detected by high-resolution tilling array, and 66 are classified as novel ones [23]. While using cDNA or EST sequences, a large number of long npcRNAs are detected in *Drosophila*, mouse and *Arabidopsis *[12,18,24-26].

Up to date, identification of long npcRNAs is limited to a few plant species, such as Arabidopsis, rice and maize. To our best knowleage, in wheat no systematic identification of long npcRNAs is reported. Wheat (*Triticum aestivum*, AABBDD, 2n = 42) is the most widely grown crop plant, occupying 17% of all the cultivated land, provides approximately 55% of carbohydrates for world human consumption [27], Biotic and abiotic stresses are important limiting factors for yield and grain quality in wheat production. For instance, powdery mildew, caused by the obligate biotrophic fungus *Blumeria graminis f. sp. tritici *(*Bgt*), is one of the most devastating diseases of wheat in China and worldwide and causing significant yield losses [28]. High temperature, often combined with drought stress, causes yield loss and reduces the grain quality [29]. To reduce the damages caused by biotic and abiotic stresses, plants have evolved sophisticated adaptive response mechanisms to reprogram gene expression at the transcriptional, post-transcriptional and post-translational levels [30]. Recently, transcript profiling has been successfully employed to determine the transcriptional responses to powdery mildew infection and heat stress in wheat, and the results revealed that a number of genes were significantly induced or repressed in response to these stresses [31,32].

In our previous study [33], it was demonstrated that expression of microRNAs in wheat was regulated by powdery mildew infection and heat stress, which stimulated us to explore whether long npcRNA was also responsive to powdery mildew infection and/or heat stress. In this study, we performed a genome-wide *in silico *screening of powdery mildew infection and heat stress responsive wheat transcripts in order to isolate a collection of long npcRNA genes. Combining microarray analysis and high-throughput SBS sequencing methods, we totally characterized 125 putative stress responsive long npcRNAs in wheat, four of them were miRNA precursors, and one was experimentally verified by northern blot. Wheat long npcRNAs displayed tissue-specific expression patterns and their expression levels were altered in response to powdery mildew infection and/or heat stress, which suggested that at least a subset of these newly identified wheat long npcRNAs potentially play roles in response to biotic and/or abiotic stresses in wheat.

## Results

### Identification of powdery mildew infection and heat stress responsive long npcRNA candidates in wheat

**Figure 1:**
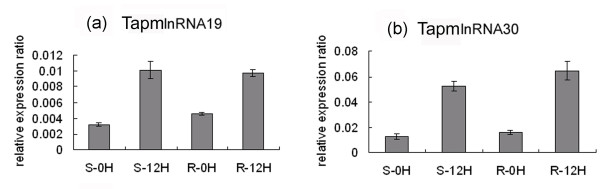
**Expression patterns of wheat long npcRNAs TapmlnRNA19 (a) and TapmlnRNA30 (b) in response to powdery mildew inoculation (12hai) as determined by qRT-PCR analysis, S-0H: before *Bgt *inoculation in susceptible (S) genotype, S-12H: 12 hrs after *Bgt *inoculation in S genotype, R-0H: before *Bgt *inoculation in resistant (R) genotype, R-12H: 12 hrs after *Bgt *inoculation in R genotype**.

**Figure 2:**
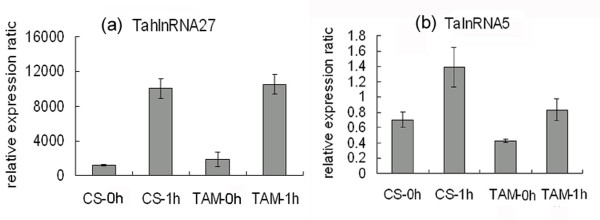
**Expression patterns of wheat long npcRNAs TahlnRNA27 (a) and TalnRNA5 (b) in response to heat stress**. CS-0h: before heat stress treatment for heat susceptible genotype Chinese Spring (CS), CS-1h: after 1 hour heat stress treatment, TAM-0h: before heat stress treatment for heat tolerant genotype TAM107 (TAM), TAM-1h: after 1 hour heat stress treatment.

In our previous study, a total of 9744 powdery mildew infection and 6560 heat stress responsive transcripts were obtained (with a fold change of at least 2) through microarray analysis using the wheat Affymetrix GeneChip^®^. In this study, in order to identify the putative wheat long npcRNAs which were responsive to powdery mildew and/or heat stress, these stress responsive transcripts were used to characterize the wheat long npcRNAs. Firstly, these transcripts were annotated by Harvest program, and 7746 and 5754 transcripts were identified to be protein-coding genes and therefore were discarded in further analysis. The remaining transcripts were then analyzed by Blastx and Blastn, 586 and 406 ESTs with no similarity to protein coding genes or tRNA and rRNA were retained. Secondly, 125 transcripts with no or short ORFs (less than 80aa) and polyA-tails were selected as putative long npcRNAs (Additional file 1), among which 71 were responsive to powdery mildew infection, and 77 were responsive to heat stress. We found that 23 long npcRNAs responded to both powdery mildew infection and heat stress (designated TalnRNA). A total of 48 putative long npcRNAs were only responsive to powdery mildew infection (designated TapmlnRNA), and 54 were only responsive to heat stress (designated TahlnRNA). Among these putative long npcRNAs, the longest ORF was 74aa, with an average of 43.5aa (Additional file 1). In order to validate expression patterns of the long npcRNAs in response to powdery mildew infection and/or heat stress, expression patterns of 4 long npcRNAs, TapmlnRNA19, TapmlnRNA30, TahlnRNA27 and TalnRNA5, were determined by using quantitative RT-PCR analysis. Expression levels of TapmlnRNA19 and TapmlnRNA30 were up-regulated after powdery mildew inoculation (Figure 1a, b), whereas expression of TahlnRNA27 and TalnRNA5 were up-regulated after heat stress (Figure 2a, b), which showed consistent expression patterns with microarray analysis.

### Four long npcRNA transcripts correspond to miRNA precursors

By mapping miRNAs which were identified from our previously sequenced six small RNA libraries (S-0h, S-12h, R-0h, R-12h, TAM-0h, TAM-1h) [33] to the complete collection of 125 long npcRNAs, we identified that four transcripts (TalnRNA5, TapmlnRNA8, TapmlnRNA19, TahlnRNA27) were miRNA precursors. Prediction of the secondary structure for the four transcripts by using the Vienna RNA package RNAfold web interface program showed that these four miRNA precursors had stable hairpin structures (Additional file 2, 3, 4 and 5).

**Figure 3:**
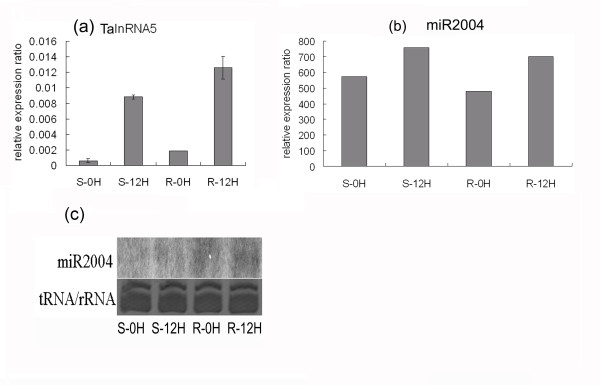
**Expression pattern of wheat long npcRNA TalnRNA5 and its corresponding miRNA before or 12hai in both disease resistant genotype (R) and susceptible genotype (S)**. (a) The expression level of TalnRNA5 as determined by qRT-PCR. (b) The expression pattern of miR2004 based on high throughput sequencing. (c) Northern blot analysis for miR2004 expression before or 12hai in S genotype and R genotype.

Among the four long npcRNAs, three (TalnRNA5, TapmlnRNA19 and TapmlnRNA8) were responsive to powdery mildew infection. Both TalnRNA5 and TapmlnRNA19 were the precursors of miR2004, and TapmlnRNA8 was the precursor of miR2066. It is interesting to note that TapmlnRNA19 and TalnRNA5 were up-regulated after powdery mildew infection as determined by qRT-PCR (Figure 1a, 3a), and miR2004 was also found to be up-regulated based on the small RNA high throughput sequencing (Figure 3b). To further determine the expression pattern of miR2004, we performed Northern blot analysis (Figure 3c) which indicated that miR2004 shared similar expression pattern with the high throughput sequencing.

The heat responsive long npcRNA TahlnRNA27 contained Ta-miR2010 family sequences, and was up-regulated in 'TAM107' (heat tolerant cultivar) 1 h after heat treatment (Figure 2a), whereas Ta-miR2010 was also statistically up-regulated 1 h after heat stress in the small RNA databases of 'TAM107' in our previous study [33]. The secondary structure and the corresponding expression pattern indicated that TahlnRNA27 might be the precursor of miR2010. In addition, the powdery mildew infection responsive long npcRNA TalnRNA5 (Figure 3a) was found to be also responsive to heat stress and the expression level was increased in 'CS' and 'TAM107' 1 h after heat stress (Figure 2b).

### Characterization of putative long npcRNAs for siRNA

**Figure 4:**
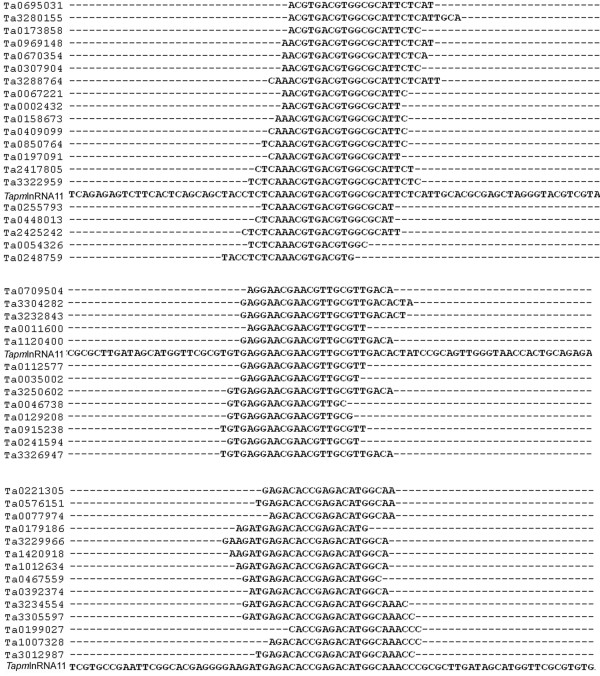
**The positions of siRNAs matching to the TapmlnRNA11**.

**Figure 5:**
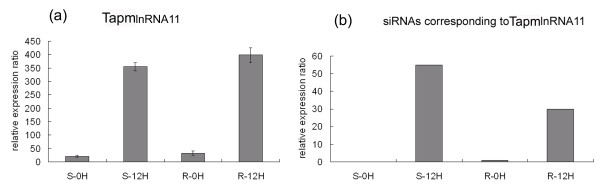
**Expression patterns of wheat long npcRNAs and their corresponding siRNAs before or 12hai in S genotype and R genotype**. (a) The expression pattern of TapmlnRNA11 in wheat microarray analysis. (b) The abundance of corresponding siRNAs matching TapmlnRNA11 based on high-throughput sequencing.

We found that 16 out of 71 powdery mildew responsive long npcRNAs gave rise to small RNAs (Additional file 1), and all of them had similar expression pattern in microarray analysis and SBS sequencing. Most of these long npcRNAs produced more than one small RNA family. For example, TapmlnRNA11 comprised three small RNA family sequences and each had several members (Figure 4). The expression level of TapmlnRNA11 in non-inoculated genotypes was quite low, but accumulated to a high level after powdery mildew infection in JD8 and JD8-Pm30 12hai (Figure 5a). Consistent with this expression pattern, its corresponding siRNAs were also up-regulated after powdery mildew infection (Figure 5b) in both genotypes.

**Figure 6:**
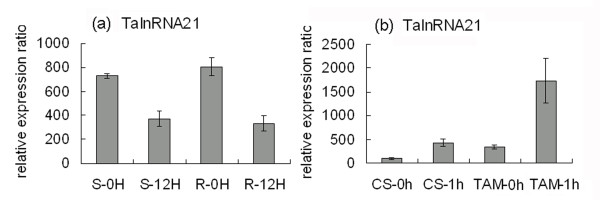
**The expression pattern of TalnRNA21 in response to powdery mildew inoculation (a) and heat stress (b) based on microarray analysis**.

For the heat stress responsive long npcRNAs, there were nine transcripts matching the small RNAs (Additional file 1). Among them, TalnRNA21 was responsive to both heat treated and powdery mildew inoculated wheat leaves, however, the expression pattern was quite different, expression of TalnRNA21 was repressed in JD8 and JD8-Pm30 12hai (Figure 6a), but up-regulated after heat stress in 'CS' and 'TAM107' (Figure 6b). We also noted that TalnRNA21 accumulated to a much higher expression level 1 h after heat treatment in heat tolerant cultivar as compared to that in heat sensitive cultivar (Figure 6b).

### Long npcRNAs corresponding to SRP and snoRNAs

**Figure 7:**
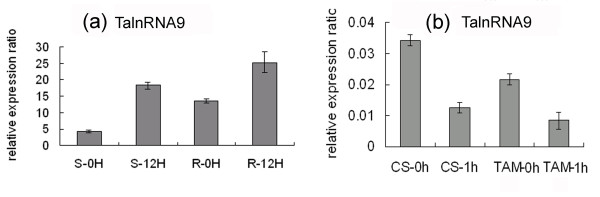
**The expression patterns of TalnRNA9 in response to powdery mildew inoculation (a) and heat stress (b) as determined by qRT-PCR**.

**Figure 8:**
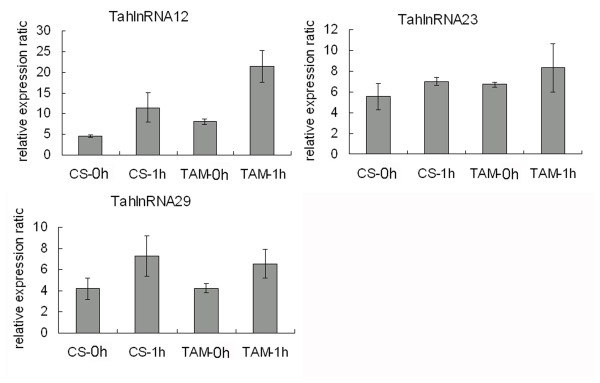
**The expression patterns of TahlnRNA12, TahlnRNA23 and TahlnRNA29 1 h after heat stress in heat sensitive genotype ('CS') and heat tolerant genotype ('TAM107') based on microarray analysis**.

We found that 52 powdery mildew infection responsive and 66 heat stress responsive long npcRNAs could execute their functions in the form of long molecules, among which 21 transcripts were responsive to both stress treatments (Additional file 1). Two transcripts, TalnRNA9 and TalnRNA12, were identified as signal recognition particle (SRP) 7S RNA variant 1 and 3, respectively. It was found that the expression of TalnRNA9 was increased in both JD8 and JD8-Pm30 genotypes 12 hours after infection (hai) (Figure 7a), but was repressed 1 h after heat treatment in 'CS' (heat sensitive cultivar) and 'TAM107' (heat tolerant cultivar) (Figure 7b). Among the 45 long npcRNAs which were only responsive to heat stress, three (TahlnRNA12 TahlnRNA23 and TahlnRNA29) were characterized as U3 snoRNAs, and their expression levels were increased 1 h after heat stress in both 'CS' and 'TAM107'(Figure 8)

### Histone acetylation of TalnRNA5 and TapmlnRNA19

**Figure 9:**
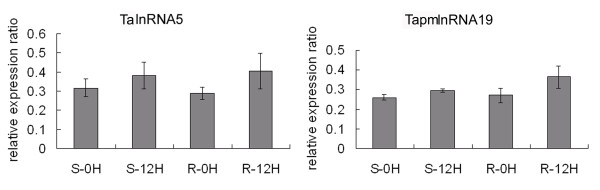
**The H3K9 acetylation levels of TalnRNA5 and TapmlnRNA19 in S and R genotypes before or 12 hrs after powdery mildew inoculation as determined by qRT-PCR**.

The histone acetylation levels of TalnRNA5 and TapmlnRNA19 were detected using antibody H3K9 by ChIP according to the procedure of Lawrence [34]. ChIP analysis indicated that acetylation levels of TalnRNA5 and TapmlnRNA19 in the inoculated JD8 and JD8-Pm30 increased as compared to the non-inoculated controls (Figure 9).

### Small RNAs might influence long npcRNAs expression

**Figure 10:**
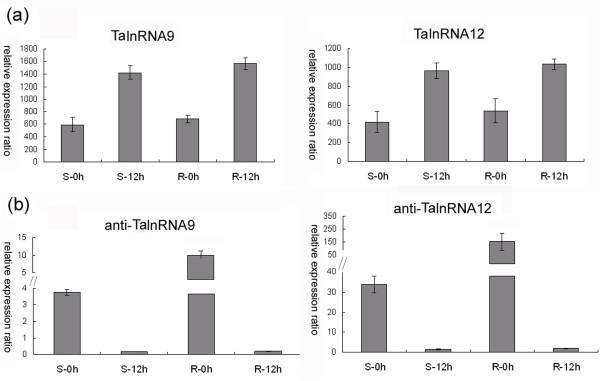
**Expression patterns of sense and antisense sequences for TalnRNA9 and TalnRNA12 before or 12 hrs after *Bgt *inoculation in S genotype and R genotype**. (a) Expression patterns of sense sequences revealed by microarray analysis. (b) Expression patterns of antisense sequences as determined by qRT-PCR.

Based on our analysis, two SRP 7S RNA variants TalnRNA9 and TalnRNA12 could be regulated by 24 nt siRNAs. There were five siRNA families complementarily matching to the long npcRNAs, among which, three groups (group I, group II, group III) matched both TalnRNA9 and TalnRNA12, and other two (group IV group V) were specific for TalnRNA9 (Additional file 6). We designed gene specific primers (Additional file 7) and amplified the antisense strand sequences of TalnRNA9 and TalnRNA12 (anti-TalnRNA9 and anti- TalnRNA12). It was found that expression levels of TalnRNA9 and TalnRNA12 were up-regulated after powdery mildew inoculation in the two genotypes (Figure 10a), whereas both of the antisense sequences were down-regulated after powdery mildew inoculation in the two genotypes (Figure 10b), and negative correlation in expression levels was observed between sense strand and antisense strand expression patterns in both JD8 and JD8-Pm30 (Figure 10). In addition, three long npcRNAs, TapmlnRNA11, TapmlnRNA41 and TapmlnRNA42 also had several group small sequences matching them, and their expression patterns could be also regulated by siRNAs.

### Wheat putative long npcRNAs displayed tissue-specific expression patterns

**Figure 11:**
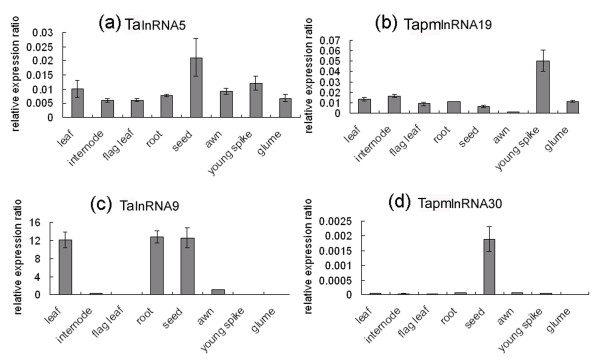
**Expression patterns of TalnRNA5, TapmlnRNA19, TalnRNA9 and TapmlnRNA30 in eight tissues as determined by qRT-PCR**.

To investigate the expression patterns of long npcRNAs in different wheat tissues, qRT-PCR was performed in 8 wheat tissues using gene specific primer pairs (Additional file 7), including leaf, internode, flag leaf, root, seed, awn, young spike and glume (Figure 11).

It was found that wheat long npcRNAs displayed tissue-specific expression patterns. TapmlnRNA30 was only detected in seed, whereas TapmlnRNA19 accumulated preferentially in young spike (Figure 11). TalnRNA5 was expressed in all the tissues, but expression level was relatively higher in seed as compared to other tissues (Figure 11). TalnRNA9 was abundantly expressed in leaf, root and seed, no signal was detected in other tissues (Figure 11). Interestingly, although both TalnRNA5 and TapmlnRNA19 gave rise to miR2004, their expression patterns were obviously different (Figure 11). In addition, TalnRNA9 was expressed quite differently between leaf and flag leaf, and the transcripts accumulated predominantly in leaf (Figure 11).

### Experimentally verified full length cDNA of predicted long npcRNAs

**Figure 12:**
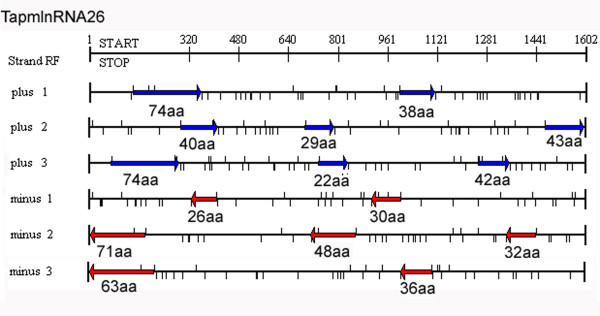
**The 15 short possible open reading frames (ORFs) positioned in TapmlnRNA26**.

In order to obtain the full length cDNAs corresponding to the long npcRNAs, we performed 5'RACE for four long npcRNAs, including TapmlnRNA26, TalnRNA21, TahlnRNA37 and TahlnRNA47. The cDNA from young leaf of JD8 was amplified by using gene specific primers (Additional file 7) and sequenced. The full length cDNAs corresponding to TapmlnRNA26, TalnRNA21, TahlnRNA37 and TahlnRNA47 were 1599 bp, 1497 bp, 737 bp and 988 bp in length, respectively. The ORFs of these sequences were searched by using ORF finder program, and no ORFs longer than 80aa was found in these full length cDNAs (Additional files 8, 9 and 10). For example, TapmlnRNA26 contained 15 putative ORFs, but the longest ORF was only 74aa (Figure 12).

## Discussion

### Wheat long npcRNAs are not conserved among the plant species and responsive to both biotic and abiotic stresses

By using combination of microarray and SBS sequencing, a total of 125 putative long npcRNAs were identified in wheat leaves using strict criteria across a collection of more than 9700 powdery mildew and 6500 heat stress responsive sequences. Our analysis could fail to identify the *bona fide *long npcRNAs in wheat due to the limited genomic information and gene annotation of wheat, however, these 125 putative long npcRNAs constituted a reliable set of wheat long npcRNAs. It must be pointed out that, in the absence of wheat whole genomic information and the full length sequences of these wheat long npcRNAs, some of them might turn out to be protein-coding RNAs when the wheat genomic sequences are available. However, this study represents the first attempt to characterize the wheat long npcRNAs and their responses to biotic and/or abiotic stresses, which could provide a starting point for further investigation of long npcRNAs in wheat.

As most non-protein coding RNAs were subjected to a low degree of evolutionary constraint, we found that the 125 long npcRNAs identified in this study had no homologs or significant matches out of plant, animal and microorganism kingdoms, and were wheat specific except for two SRP 7SRNA variants (TalnRNA9 and TalnRNA12) and 3 U3 snoRNAs (TahlnRNA12 TahlnRNA23 and TahlnRNA29), which was in good agreement to the previous studies in other species such as *Drosophila*, *Arabidopsis *and mouse [12,25,26]. Also, these long npcRNAs did not appear to form large homologous family. This might suggest that during the evolution, wheat had developed a batch of specific long npcRNAs to regulate gene expression and cell activity. Further analysis revealed that long npcRNAs in wheat had tissue-specific expression patterns, similar expression patterns of long npcRNAs were also reported in other species [24-26]. In our investigation, even in leaf and flag leaf, TalnRNA9 was differentially expressed, which suggested that long npcRNAs probably had much more precise expression regulation mechanisms. In addition, though TalnRNA5 and TapmlnRNA19 gave rise to the same miRNA, they displayed distinct expression patterns, indicating that miRNA could potentially be produced by different precursors in different wheat tissues.

SRP RNA is an exception, as it is a ribonucleoprotein (protein-RNA complex) that recognizes and targets specific proteins to the endoplasmic reticulum in eukaryotes and the plasma membrane in prokaryotes. Moreover, U3 snoRNAs predominantly found in the nucleolus are thought to guide site-specific cleavage of ribosomal RNA (rRNA) during pre-rRNA processing. Therefore, they are thought to be conserved across three kingdoms.

It was reported that increased expression of either BC200 or an antisense transcript of the b-secretase-1 (BACE1) gene had been implicated in the progression of Alzheimer's disease [35,36]. Ben et al [18] found that abiotic stress altered the accumulation of 22 out of the 76 npcRNAs. These indicated that long npcRNAs had been linked to biotic and/or abiotic stresses, though in most instances, evidence had relied on differences in transcript expression levels between treated and non-treated samples. Our analysis added further evidence for the responsiveness of long npcRNAs to both biotic and/or abiotic stresses, since 71 wheat long npcRNAs were responsive in defense against powdery mildew infection, and 77 were responsive to heat stress.

### Some of the wheat long npcRNAs are small RNA precursors

Study showed that miR675 was derived from the long npcRNA H19 which was endogenously expressed in human keratinocytes and neonatal mice [37], and .npcRNA78 gene contained the miR162 sequence in an alternative intron and corresponded to the MIR162a locus [24]. The 'BIC' noncoding RNA that served as the precursor for miR155 was also readily detectable *in vivo *as full-length transcripts [38]. In our identified wheat long npcRNAs, four transcripts (TalnRNA5, TapmlnRNA8, TapmlnRNA19, and TahlnRNA27) were characterized as putative miRNA precursors. Among them, TapmlnRNA8, TapmlnRNA19 were specific to powdery mildew infection, while TahlnRNA27 was only responsive to heat stress. Increasing evidence indicated that miRNAs played important roles in plant responses to biotic stresses [9,39,40]. After powdery mildew infection, TalnRNA5 and TapmlnRNA19 were up-regulated 12hai in JD8 and JD8-Pm30 genotypes, and their corresponding miR2004 was also increased in abundance, which strongly indicated that these two long npcRNAs were processed to miRNAs to regulate wheat response to powdery mildew infection. However, as there were no significant expression differences between the NILs JD8 and JD8-Pm30, we speculated that these two long npcRNAs functioned as basal defense. To further confirm this hypothesis, TalnRNA5, TapmlnRNA19, TalnRNA9 and TapmlnRNA30 were analyzed using qRT-PCR in 12 hrs after-touched JD8 and JD8-Pm30 as well as their controls, and their expression level had no differences between two treatments (data not show), which suggested that the expression alteration were caused by powdery mildew infection, not by touching.

In addition, we revealed that 26 wheat long npcRNAs produced siRNAs and 97 sequences could function in the form of long molecules involved in wheat resistance to powdery mildew infection and/or heat stressed. The collection of long npcRNAs offered candidates for further analysis of this kind of npcRNAs, which gained increasing attention in recent years [9,41,42].

Our analysis revealed that two SRP 7S RNA variants (TalnRNA9 and TalnRNA12) as well as TapmlnRNA11, TapmlnRNA41 and TapmlnRNA42 could be regulated by siRNAs. Coram et al [43] reported that the antisense strands of probe sets Ta.21480 and Ta.24771 (namely, TalnRNA9 and TalnRNA12) were expressed using wheat Affymetrix genome array, which was in good agreement with our experimental results. And interestingly, our analysis also shown that the expression patterns of antisense had negative correlations with sense sequence for both TalnRNA9 and TalnRNA12, which strongly indicated that the siRNAs generated from antisense strands might regulate expression of their corresponding sense strands. Collectively, this study indicated that expression of wheat long npcRNAs might be regulated by other non-protein coding RNAs, as was the case for *Xist *gene [44].

## Conclusion

In summary, by using computational analysis and experimental approach, for the first time, we identified 125 putative wheat long npcRNAs. These identified wheat long npcRNAs were not conserved among plant species, and some of them were small RNA precursors. Wheat long npcRNAs showed a tissue dependent expression patterns and their expressions were responsive to powdery mildew infection and/or heat stress, suggesting that they could play roles in development and regulation of biotic and/or abiotic stresses. Our analysis also indicated that expressions of some wheat long npcRNAs could be regulated by small RNAs and through histone acetylation, but this need further investigation. The identification and expression analysis of wheat long npcRNAs in this study would provide a starting point to understand their functions and regulatory mechanisms in the future.

## Methods

### Plant materials

Seeds of powdery mildew susceptible wheat cultivar 'JD8' (designated as S) and its near isogenic line carrying a powdery mildew resistance gene *Pm30 *(designated as R) were planted in 8-10 cm diameter pots. Seedlings were artificially inoculated when the first leaf was fully expanded, with a local prevalent *Blumeria. graminis *f. sp. *tritici *isolate E09. Inoculation was performed by dusting or brushing conidia from neighboring sporulating susceptible seedlings onto the test seedlings. Leaf samples were collected from both genotype at 0 and 12 hrs post inoculation (designated as S-0 h, S-12 h, R-0 h, R-12 h), respectively, and frozen in liquid nitrogen and used for RNA extraction.

For heat stress, heat tolerant genotype 'TAM107' was used in this study. Seeds were surface-sterilized in 1% sodium hypochlorite for 15 min, rinsed in distilled water, and soaked in dark overnight at room temperature. The germinated seeds were transferred into the pots (25 seedlings per pot) containing vermiculite. The treatments were carried out as described by Qin et al [32]. Leaves were collected at 0 and 1 hour after heat treatment (designated TAM-0 h and TAM-1 h) At the end of heat treatments, the leaves were frozen in the liquid nitrogen immediately, and then stored at -80°C for further use.

### Microarray analysis

Total RNA was extracted using Trizol reagent (Invitrogen) following the manufacture's recommendations. Briefly, mRNA was enriched from 80~90 μg total RNA using the RNeasy Plant Mini Kit (QIAGEN) according to the protocol, and was subsequently reverse-transcribed to double stranded cDNA using the GeneChip^® ^Two-Cycle cDNA Synthesis Kit. The biotin labeled cRNA was made using the GeneChip^® ^IVT Labeling Kit (Affymetrix, CA, USA). Twenty micrograms of cRNA samples were fragmented and hybridized for 16 h at 45°C to the Affymetrix Wheat Genome Array (Santa Clara, CA, USA). After washing using the Genechip^® ^Fluidics Station 450, arrays were scanned using the Genechip^® ^3000 Scanner that is located in Bioinformatics Center at China Agriculture University (NCBI accession Number: GSE27339 http://www.ncbi.nlm.nih.gov/projects/geo/query/acc.cgi?acc=GSE27339).

### Small RNA sequencing

Small RNA libraries (S-0 h, S-12 h, R-0 h, R-12 h, TAM-0 h, TAM-1 h) preparation and sequencing were performed with Solexa sequencing technology (BGI, Shenzhen, China) as described by Sunkar et al [45]. Automated base calling of the raw sequence and vector removal were performed with PHRED and CROSS MATCH programs [46,47]. Trimmed 3' and 5' adapters sequences, removed RNAs less than 17 nt and polyA, only sequences longer than 17 nt with a unique ID were used for further analysis. We calculated sequencing frequency of each small RNA sequence, the number of reads for each sequence reflecting relative abundance (NCBI accession Number: GSE27339, http://www.ncbi.nlm.nih.gov/projects/geo/query/acc.cgi?acc=GSE27339).

### Computational methods for long npcRNA identification

**Figure 13:**
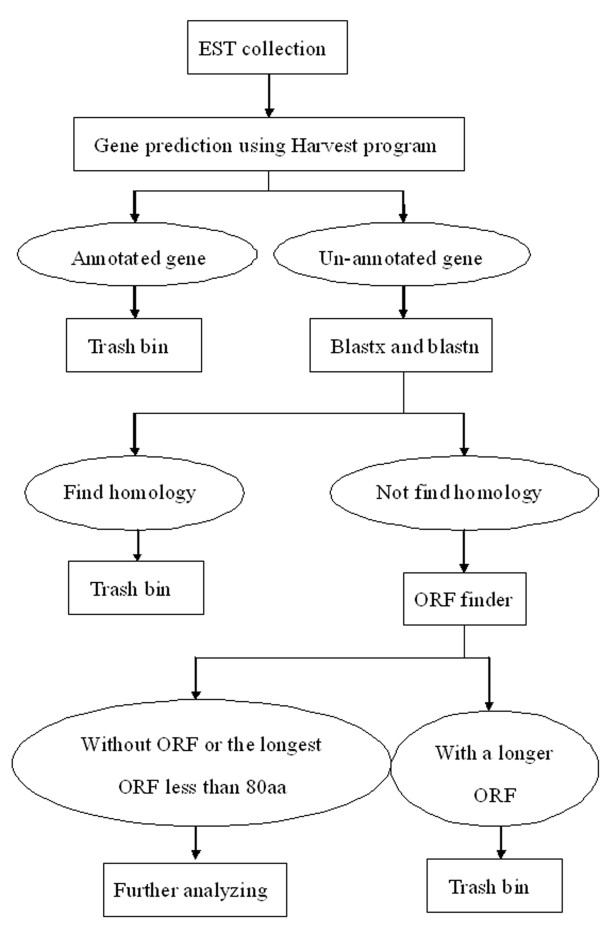
**Schematic representation of computational method for long npcRNA identification**.

Firstly, powdery mildew infection and heat stress responsive transcripts were annotated by Harvest program, and protein-coding genes are discarded, the reminding sequences were analyzed by Blastx and Blastn, and ESTs with no similarity to protein coding genes or tRNA and rRNA were retained. Secondly, we screened the reminding ESTs, and the sequences with polyA-tail were selected. Thirdly, we predicted the longest ORF of these transcripts using ORF finder, and the sequences with no or short ORFs (less than 80aa) were retained for further analysis (Figure 13).

### Chromatin-Immunoprecipitation (ChIP) assays

ChIP was modified from published protocol [34]. Approximately 1 g of leaves was used for each ChIP assay. The fresh tissues were subjected to vacuum infiltration in formaldehyde (1%) solution for cross-linking the chromatin proteins to DNA. Chromatin was extracted and sonicated for 4 × 10 sec pulses, 40% duty cycle and 20% power with chilling on ice for 1 min after each pulse. The average size of the resulted DNA fragments ranged between 0.2~2.0-kb, centering around 500 bp. An aliquot of chromatin solution (1/10 of total volume) was used to determine the DNA fragment sizes and serve as input control. The remaining chromatin solution was diluted 10-fold and divided into two aliquots. One aliquot was incubated by adding 10 μl of antibodies (anti-acetyl-histone H3K9, Upstate Biotechnology, NY). The other aliquot was incubated without antibodies (mock). After incubation at 4°C with rotation for overnight, the solution was added to 40 μl of protein A agarose and incubated for another 1 hours. The immunocomplexes were eluted and crosslinks were reversed by incubation at 65°C for 15 min. Residual protein was degraded by proteinase K and DNA was extracted and dissolved in 50 μl of ddH_2_O.

### Quantitative Real-Time PCR (qRT-PCR) Analysis

qRT-PCR was performed using the ChIP DNA or cDNA samples in a 10-μl mixture containing 1× LightCycler-FastStart DNA master SYBR Green I. According to MIQE guidelines, qRT-PCR was performed as follows: initial denaturation for 10 min at 95°C, followed by 40 cycles of 30 s at 95°C, 45 s at 55 to 60°C, 10 s at 72°C, and 72°C for 5 min as the last step. The threshold cycles (Ct) of each test target were averaged for triplicate reactions and the values were normalized according to the Ct of the control products (*Ta*-actin).

## Authors' contributions

MX, YW, YY and DQ carried out the microarray analysis and small RNA sequencing, participated in the long npcRNA identification, and draft the manuscript. MX and ZH carried out ChIP analysis, NS, CX and HP carried out the qRT-PCR analysis. ZN and QS carried out the design of the study and finish the manuscript. All authors read and approved the final manuscript.

## Supplementary Material

Additional file 1 **Wheat long npcRNAs responsive to powdery mildew infection and/or heat stress**. The table includes lnpcRNAs' ID, probe set ID, the longest ORF, the number of putative ORFs and corresponding siRNAs. Click here for file

Additional file 2 **The hairpin structure of putative TahlnRNA27**. The figure shows the secondary structure of putative wheat long npcRNA TahlnRNA27 by using the Vienna RNA package RNAfold web interface program, the perfect hairpin structure indicates that it might give rise to miRNA. Click here for file

Additional file 3 **The hairpin structure of putative TalnRNA5**. The figure shows the secondary structure of putative wheat long npcRNA TahlnRNA5 by using the Vienna RNA package RNAfold web interface program, the perfect hairpin structure indicates that it might give rise to miRNA. Click here for file

Additional file 4 **The hairpin structure of putative TalnpmRNA8**. The figure shows the secondary structure of putative wheat long npcRNA TahlnRNA8 by using the Vienna RNA package RNAfold web interface program, the perfect hairpin structure indicates that it might give rise to miRNA. Click here for file

Additional file 5 **The hairpin structure of putative TalnpmRNA19**. The figure shows the secondary structure of putative wheat long npcRNA TahlnRNA19 by using the Vienna RNA package RNAfold web interface program, the perfect hairpin structure indicates that it might give rise to miRNA. Click here for file

Additional file 6 **Categories of siRNAs corresponding to SRP1 and SRP3 7S RNA variants and sequences of SRP1 and SRP2 corresponding siRNAs**. (a) The siRNAs corresponding to SRP1 and SRP3 7S RNA variants are categorized to 5 groups according to their locations, most members of group I, II, and III match both TalnRNA9 and TalnRNA12, and other two (group IV group V) are specific for TalnRNA9. (b) The table includes sequences of of SRP1 and SRP3 corresponding siRNAs Click here for file

Additional file 7 **Primer sequences for 5'RACE and real time PCR**. The table displays the sequences of primers used for both 5'RACE and real time PCR Click here for file

Additional file 8 **The short possible ORFs in TalnRNA21**. The figure displays all the possible ORFs in the full length cDNA of TalnRNA21, and none of them are longer than 80aa. Click here for file

Additional file 9 **The short possible ORFs in TahlnRNA37**. The figure displays all the possible ORFs in the full length cDNA of TalnRNA37, and none of them are longer than 80aa. Click here for file

Additional file 10 **The short possible ORFs in TahlnRNA47**. The figure displays all the possible ORFs in the full length cDNA of TalnRNA47, and none of them are longer than 80aa. Click here for file
