# Supplementary material for: Identification and characterization of wheat long non-protein coding RNAs responsive to powdery mildew infection and heat stress by using microarray analysis and SBS sequencing
Source: BMC Plant Biol. 2011 Apr 7;11:61. doi: 10.1186/1471-2229-11-61 (PMC3079642; doi:10.1186/1471-2229-11-61)
Supplement: Additional file 1 — Wheat long npcRNAs responsive to powdery mildew infection and/or heat stress. The table includes lnpcRNAs' ID, probe set ID, the longest ORF, the number of putative ORFs and corresponding siRNAs. [file 1471-2229-11-61-S1.DOC]

**Additional data file1: Wheat long npcRNAs responsive to powdery infection and/or heat stress**

| **ID** | **Probe set ID** | **Longest OFR(aa)** | **No.of ORF** | **No.of corresponding siRNAs** |
| --- | --- | --- | --- | --- |
| TapmlnRNA1 | Ta.11089.1.A1_at | 56 | 3 | - |
| TapmlnRNA2 | Ta.11365.1.A1_at* | 39 | 1 | 12 |
| TapmlnRNA3 | Ta.11993.1.S1_at | 49 | 2 | - |
| TapmlnRNA4 | Ta.12108.1.A1_at | 47 | 1 | - |
| TapmlnRNA5 | Ta.12148.1.S1_a_at | 61 | 2 | - |
| TapmlnRNA6 | Ta.12336.1.A1_at | 47 | 3 | - |
| TapmlnRNA7 | Ta.13279.3.S1_at* | 51 | 1 | 8 |
| TapmlnRNA8 | Ta.13303.1.S1_at# | 42 | 3 | - |
| TapmlnRNA9 | Ta.14468.1.A1_at* | 55 | 2 | 34 |
| TapmlnRNA10 | Ta.15074.1.S1_at | 36 | 1 | - |
| TapmlnRNA11 | Ta.15193.1.S1_at* | 55 | 2 | 71 |
| TapmlnRNA12 | Ta.15200.1.A1_at | 60 | 1 | - |
| TapmlnRNA13 | Ta.15934.1.S1_at | 60 | 2 | - |
| TapmlnRNA14 | Ta.1603.1.S1_at | 34 | 1 | - |
| TapmlnRNA15 | Ta.16779.1.S1_at* | 58 | 5 | 9 |
| TapmlnRNA16 | Ta.17187.1.S1_at* | 59 | 2 | 29 |
| TapmlnRNA17 | Ta.17883.1.S1_at | 44 | 1 | - |
| TapmlnRNA18 | Ta.18151.2.S1_x_at | 51 | 2 | - |
| TapmlnRNA19 | Ta.19347.1.A1_at# | 36 | 1 | - |
| TapmlnRNA20 | Ta.19370.1.S1_at | 38 | 1 | - |
| TapmlnRNA21 | Ta.20294.1.S1_at | 47 | 1 | - |
| TapmlnRNA22 | Ta.21236.2.S1_x_at | 49 | 1 | - |
| TapmlnRNA23 | Ta.21461.1.S1_at* | 38 | 1 | 21 |
| TapmlnRNA24 | Ta.22642.1.S1_at* | 43 | 2 | 10 |
| TapmlnRNA25 | Ta.22792.1.A1_at | 42 | 3 | - |
| TapmlnRNA26 | Ta.25591.1.S1_at* | 74 | 3 | 13 |
| TapmlnRNA27 | Ta.25940.1.A1_at | 52 | 2 | - |
| TapmlnRNA28 | Ta.25959.1.A1_at* | 42 | 2 | 14 |
| TapmlnRNA29 | Ta.2999.1.S1_at* | 41 | 1 | 45 |
| TapmlnRNA30 | Ta.30399.1.A1_s_at | 33 | 3 | - |
| TapmlnRNA31 | Ta.30556.1.A1_at* | 39 | 1 | 38 |
| TapmlnRNA32 | Ta.30664.1.S1_at* | 38 | 2 | 5 |
| TapmlnRNA33 | Ta.3480.2.S1_at | 35 | 3 | - |
| TapmlnRNA34 | Ta.4697.1.S1_at* | 37 | 1 | 11 |
| TapmlnRNA35 | Ta.7809.1.A1_at | 64 | 1 | - |
| TapmlnRNA36 | Ta.8362.1.A1_at | 59 | 3 | - |
| TapmlnRNA37 | Ta.8680.1.A1_at | 58 | 3 | - |
| TapmlnRNA38 | Ta.8877.1.A1_at* | 64 | 3 | 19 |
| TapmlnRNA39 | Ta.9960.1.S1_a_at | 70 | 3 | - |
| TapmlnRNA40 | TaAffx.121914.3.S1_at | 54 | 2 | - |
| TapmlnRNA41 | TaAffx.12266.1.A1_x_at | 43 | 1 | - |
| TapmlnRNA42 | TaAffx.128795.21.S1_at | 53 | 2 | - |
| TapmlnRNA43 | TaAffx.132335.1.A1_at | 68 | 4 | - |
| TapmlnRNA44 | TaAffx.15430.1.A1_at | 38 | 3 | - |
| TapmlnRNA45 | TaAffx.43051.1.S1_at | 67 | 4 | - |
| TapmlnRNA46 | TaAffx.51623.1.S1_at | 40 | 4 | - |
| TapmlnRNA47 | TaAffx.57156.1.S1_at | 33 | 2 | - |
| TapmlnRNA48 | TaAffx.67846.1.A1_at | 39 | 1 | - |
| TalnRNA1 | Ta.10864.1.S1_at | 0 | 0 | - |
| TalnRNA2 | Ta.11411.1.A1_at | 38 | 2 | - |
| TalnRNA3 | Ta.11703.1.S1_at | 57 | 2 | - |
| TalnRNA4 | Ta.12154.1.S1_at | 75 | 4 | - |
| TalnRNA5 | Ta.12786.1.A1_at# | 51 | 6 | - |
| TalnRNA6 | Ta.14899.1.S1_s_at | 42 | 1 | - |
| TalnRNA7 | Ta.15927.1.S1_at | 0 | 0 | - |
| TalnRNA8 | Ta.16175.1.A1_at | 33 | 1 | - |
| TalnRNA9 | Ta.21480.1.S1_x_at | 49 | 1 | - |
| TalnRNA10 | Ta.21750.1.S1_x_at | 47 | 1 | - |
| TalnRNA11 | Ta.24664.1.S1_at | 52 | 3 | - |
| TalnRNA12 | Ta.24771.1.S1_x_at | 36 | 1 | - |
| TalnRNA13 | Ta.27863.1.A1_at | 42 | 1 | - |
| TalnRNA14 | Ta.28771.1.A1_at | 38 | 1 | - |
| TalnRNA15 | Ta.30887.1.A1_at | 0 | 0 | - |
| TalnRNA16 | Ta.4252.1.S1_a_at | 50 | 2 | - |
| TalnRNA17 | Ta.5274.1.S1_at | 59 | 2 | - |
| TalnRNA18 | Ta.5776.1.S1_at | 42 | 1 | - |
| TalnRNA19 | Ta.8713.1.A1_at | 40 | 1 | - |
| TalnRNA20 | Ta.9029.1.A1_at | 48 | 2 | - |
| TalnRNA21 | Ta.9179.2.S1_at* | 69 | 3 | 5 |
| TalnRNA22 | TaAffx.34482.1.S1_at | 0 | 0 | - |
| TalnRNA23 | TaAffx.97191.1.S1_at | 46 | 1 | - |
| TahlnRNA1 | Ta.11539.1.A1_at | 47 | 1 | - |
| TahlnRNA2 | Ta.11651.1.A1_at | 34 | 1 | - |
| TahlnRNA3 | Ta.11725.2.S1_at* | 64 | 2 | 1 |
| TahlnRNA4 | Ta.11815.1.A1_at | 63 | 2 | - |
| TahlnRNA5 | Ta.12144.1.A1_at | 41 | 1 | - |
| TahlnRNA6 | Ta.12271.1.A1_at | 53 | 2 | - |
| TahlnRNA7 | Ta.12312.1.A1_at | 59 | 2 | - |
| TahlnRNA8 | Ta.12439.1.A1_at | 80 | 3 | - |
| TahlnRNA9 | Ta.13559.1.S1_at | 41 | 2 | - |
| TahlnRNA10 | Ta.13599.1.A1_at | 61 | 4 | - |
| TahlnRNA11 | Ta.14054.1.S1_at | 0 | 0 | - |
| TahlnRNA12 | Ta.14253.1.S1_at | 0 | 0 | - |
| TahlnRNA13 | Ta.15217.1.S1_at | 45 | 3 | - |
| TahlnRNA14 | Ta.16016.1.S1_at* | 0 | 0 | 4 |
| TahlnRNA15 | Ta.16163.1.S1_at | 34 | 2 | - |
| TahlnRNA16 | Ta.16755.1.S1_a_at | 46 | 3 | - |
| TahlnRNA17 | Ta.17695.1.A1_at | 70 | 3 | - |
| TahlnRNA18 | Ta.17860.1.A1_at | 43 | 3 | - |
| TahlnRNA19 | Ta.18066.1.S1_at* | 58 | 3 | 1 |
| TahlnRNA20 | Ta.18132.1.S1_at | 61 | 2 | - |
| TahlnRNA21 | Ta.18392.1.S1_at | 52 | 1 | - |
| TahlnRNA22 | Ta.18565.1.S1_s_at | 0 | 0 | - |
| TahlnRNA23 | Ta.19027.1.S1_at | 73 | 1 | - |
| TahlnRNA24 | Ta.19040.1.S1_at | 39 | 1 | - |
| TahlnRNA25 | Ta.1909.1.A1_at | 68 | 3 | - |
| TahlnRNA26 | Ta.19250.1.S1_s_at | 0 | 0 | - |
| TahlnRNA27 | Ta.20909.2.S1_s_at# | 39 | 3 | - |
| TahlnRNA28 | Ta.20998.1.S1_at | 67 | 2 | - |
| TahlnRNA29 | Ta.21982.1.S1_at | 43 | 3 | - |
| TahlnRNA30 | Ta.24004.1.A1_at | 45 | 2 | - |
| TahlnRNA31 | Ta.24511.1.S1_at | 41 | 3 | - |
| TahlnRNA32 | Ta.25345.1.A1_at | 36 | 1 | - |
| TahlnRNA33 | Ta.25726.1.A1_at | 52 | 3 | - |
| TahlnRNA34 | Ta.25887.1.A1_at | 42 | 1 | - |
| TahlnRNA35 | Ta.27852.1.S1_at | 43 | 4 | - |
| TahlnRNA36 | Ta.27865.1.A1_at* | 42 | 2 | 2 |
| TahlnRNA37 | Ta.28709.1.S1_at | 68 | 2 | - |
| TahlnRNA38 | Ta.28777.1.S1_s_at | 0 | 0 | - |
| TahlnRNA39 | Ta.30675.1.S1_at | 39 | 3 | - |
| TahlnRNA40 | Ta.3109.1.A1_at | 63 | 2 | - |
| TahlnRNA41 | Ta.3279.1.S1_at* | 70 | 2 | 46 |
| TahlnRNA42 | Ta.4693.1.S1_at* | 37 | 1 | 1 |
| TahlnRNA43 | Ta.6792.2.A1_at | 0 | 0 | - |
| TahlnRNA44 | Ta.7078.1.S1_at | 0 | 0 | - |
| TahlnRNA45 | Ta.7467.1.S1_x_at | 37 | 1 | - |
| TahlnRNA46 | Ta.7827.1.S1_at | 0 | 0 | - |
| TahlnRNA47 | Ta.7843.1.S1_a_at* | 36 | 1 | 1 |
| TahlnRNA48 | Ta.828.1.A1_at | 56 | 1 | - |
| TahlnRNA49 | Ta.8659.1.S1_at | 54 | 1 | - |
| TahlnRNA50 | Ta.8991.1.S1_at | 71 | 4 | - |
| TahlnRNA51 | Ta.9985.1.S1_at | 33 | 1 | - |
| TahlnRNA52 | TaAffx.113648.1.S1_at* | 0 | 0 | 2 |
| TahlnRNA53 | TaAffx.132274.1.A1_at | 0 | 0 | - |
| TahlnRNA54 | TaAffx.6443.2.S1_at | 34 | 1 | - |

# represent miRNA precursors * represent siRNA precursors
