# Supplementary figures and images for: Identification and characterization of wheat long non-protein coding RNAs responsive to powdery mildew infection and heat stress by using microarray analysis and SBS sequencing
Source: BMC Plant Biol. 2011 Apr 7;11:61. doi: 10.1186/1471-2229-11-61 (PMC3079642; doi:10.1186/1471-2229-11-61)

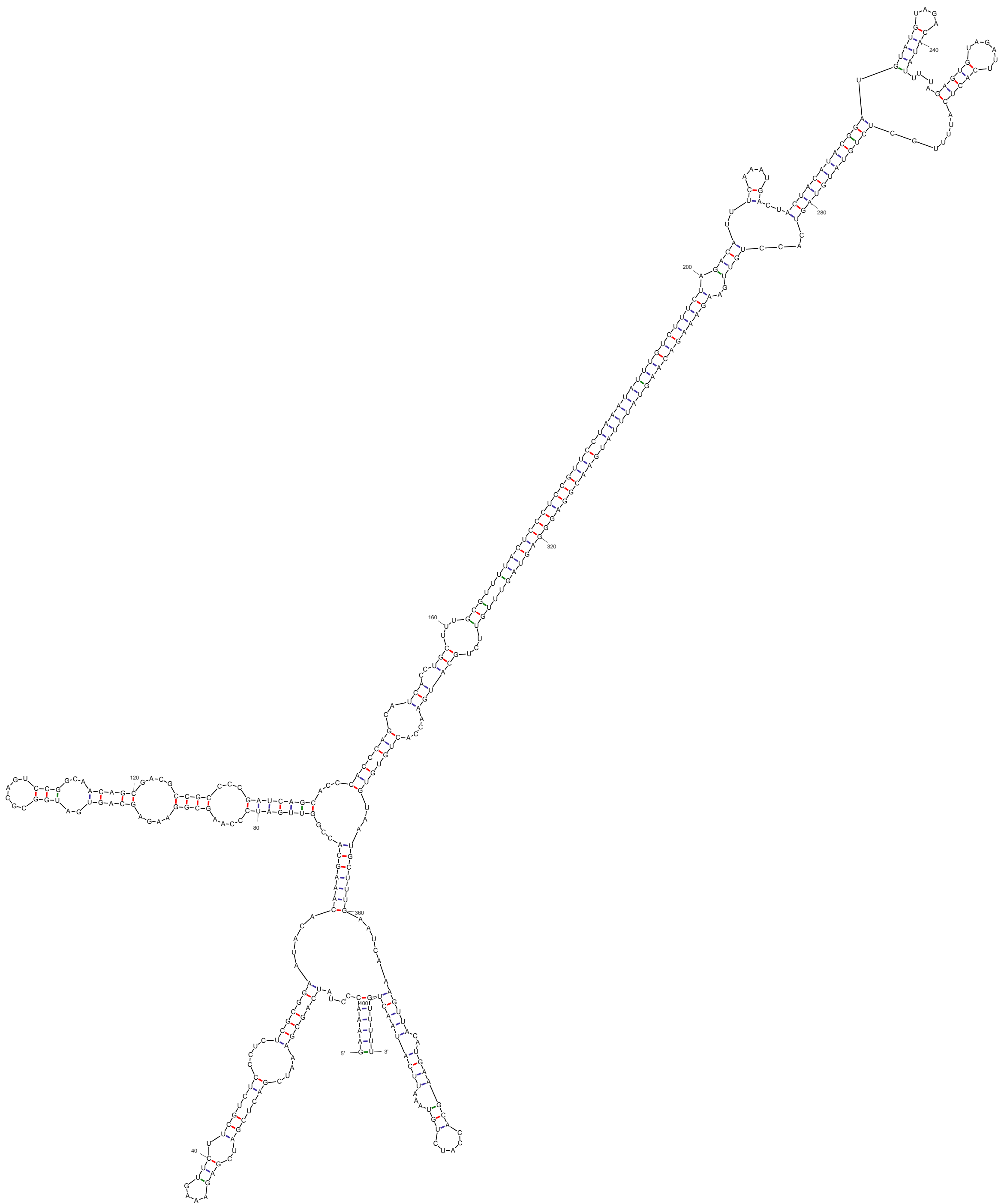

Supplement: Additional file 2 — The hairpin structure of putative TahlnRNA27. The figure shows the secondary structure of putative wheat long npcRNA TahlnRNA27 by using the Vienna RNA package RNAfold web interface program, the perfect hairpin structure indicates that it might give rise to miRNA. [file 1471-2229-11-61-S2.PDF]

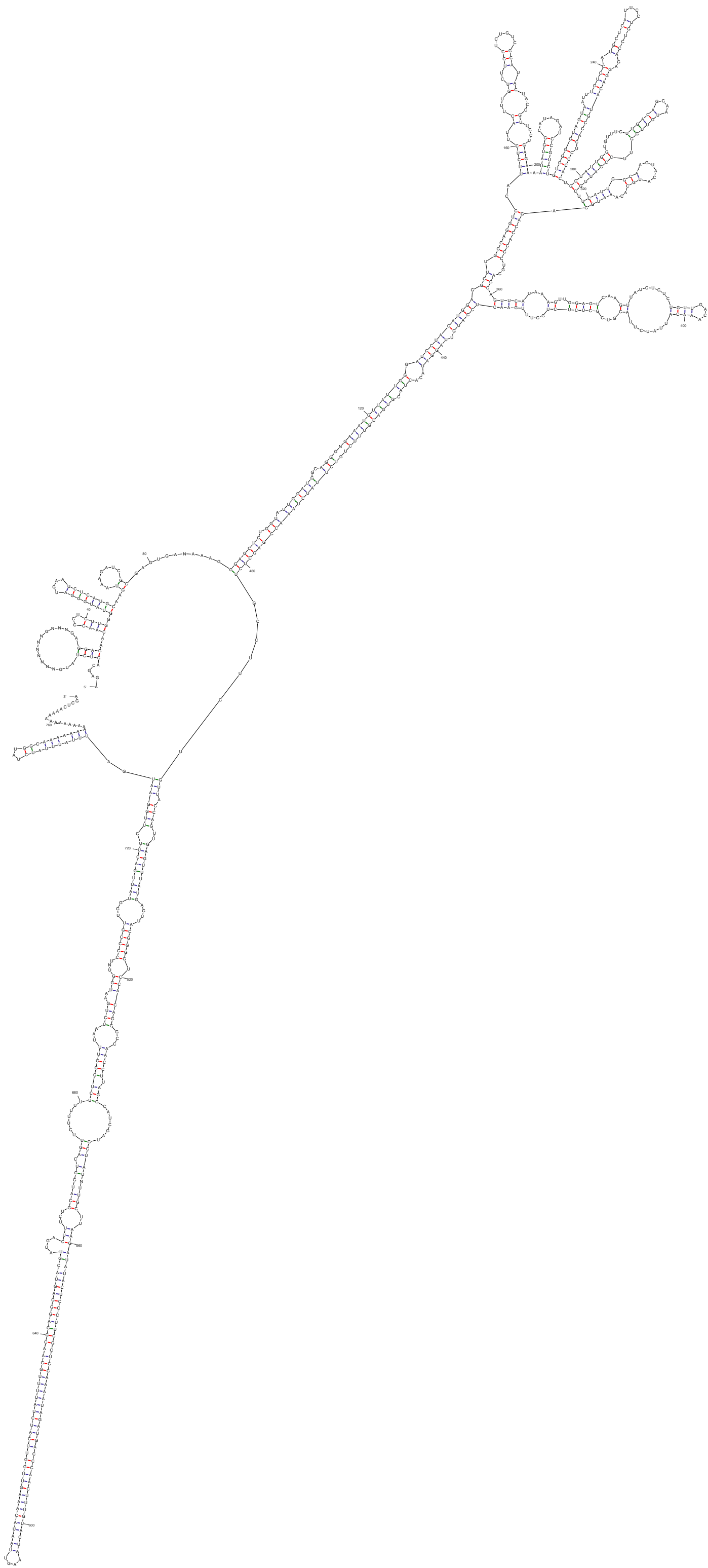

dG = -210.10 10Jan13-05-07-56

Supplement: Additional file 3 — The hairpin structure of putative TalnRNA5. The figure shows the secondary structure of putative wheat long npcRNA TahlnRNA5 by using the Vienna RNA package RNAfold web interface program, the perfect hairpin structure indicates that it might give rise to miRNA. [file 1471-2229-11-61-S3.PDF]

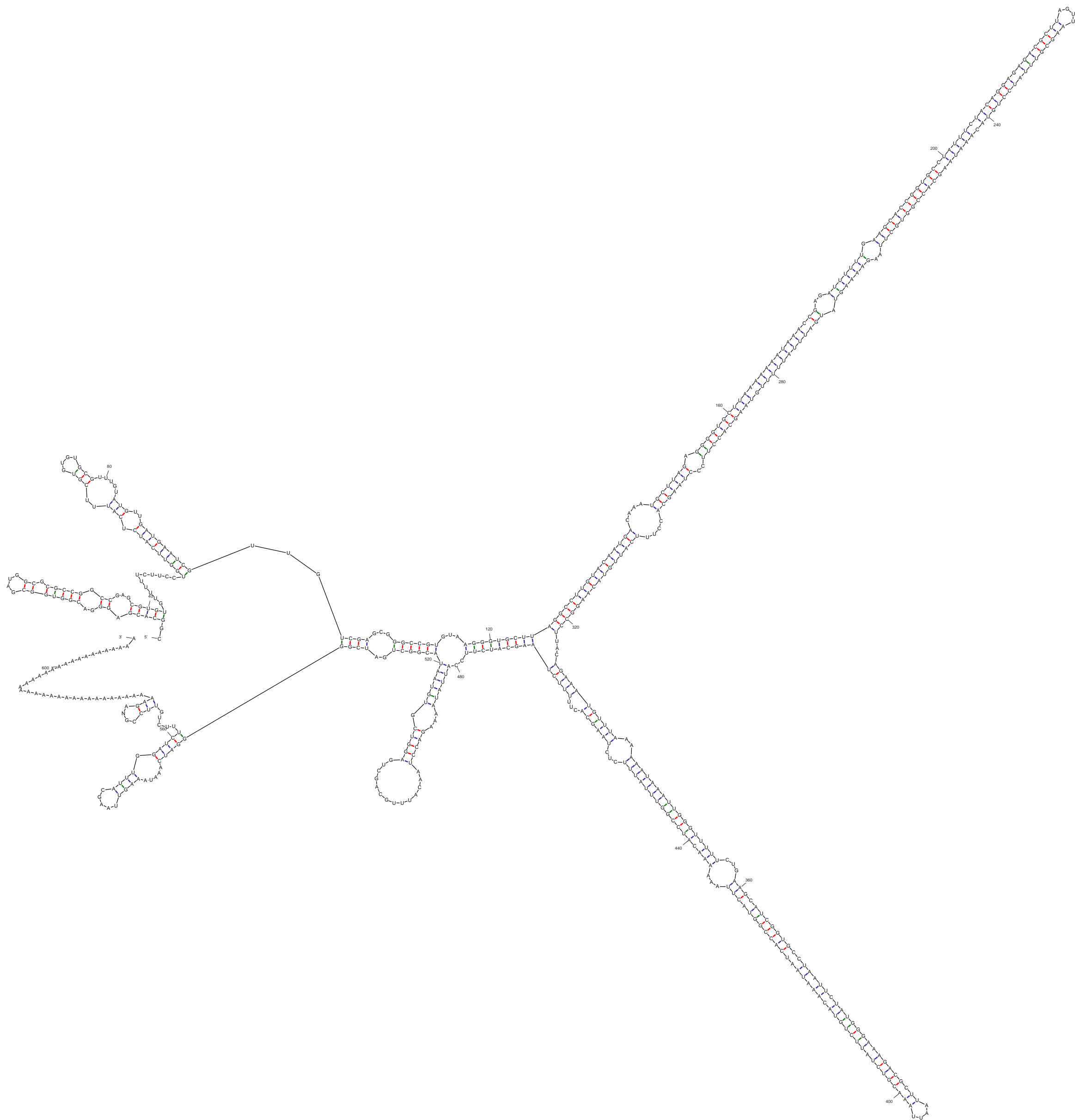

$dG = -204.70$  10Jan13-05-10-02

Supplement: Additional file 4 — The hairpin structure of putative TalnpmRNA8. The figure shows the secondary structure of putative wheat long npcRNA TahlnRNA8 by using the Vienna RNA package RNAfold web interface program, the perfect hairpin structure indicates that it might give rise to miRNA. [file 1471-2229-11-61-S4.PDF]

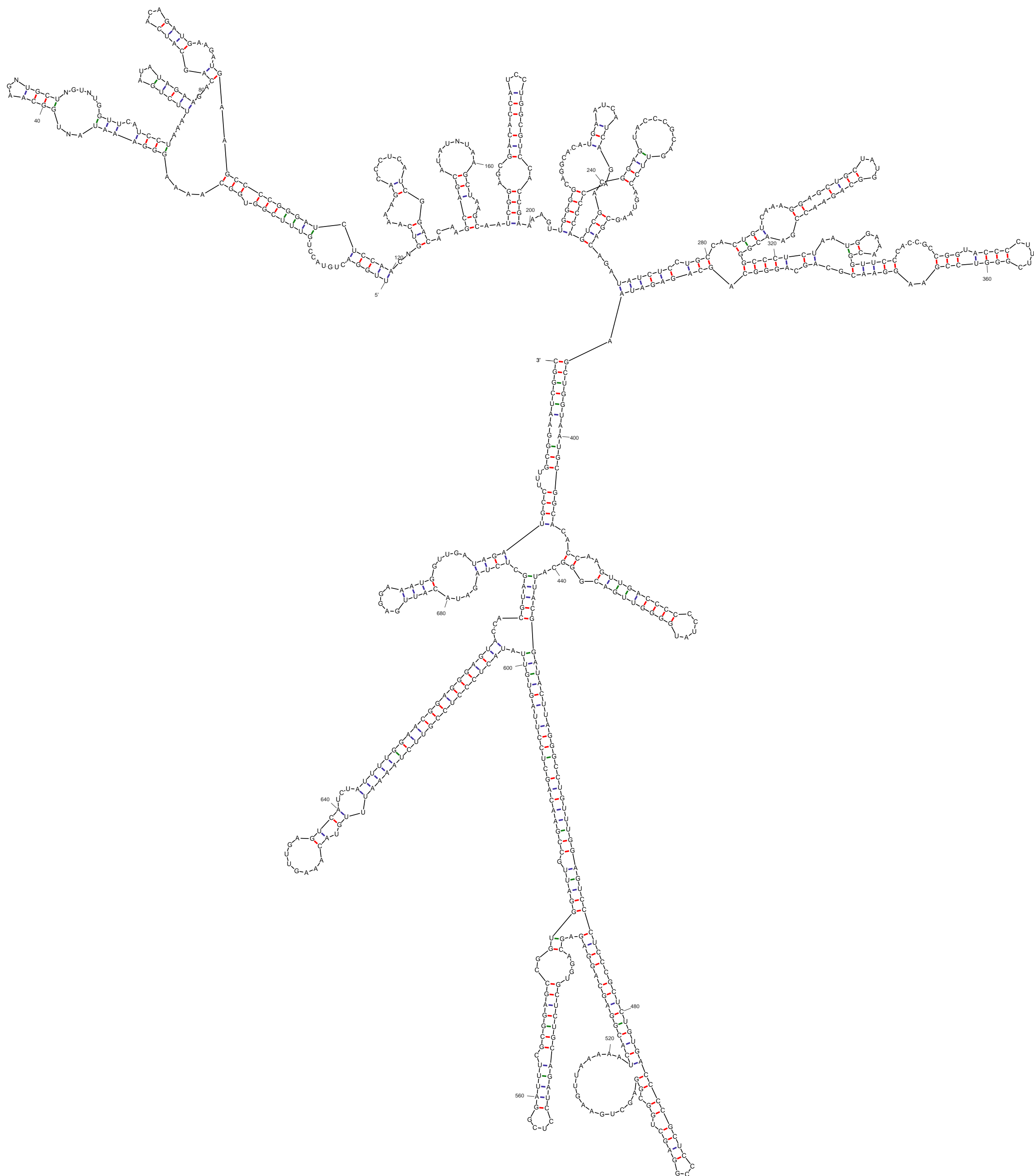

$dG = -209.80$  10Jan14-08-33-58

Supplement: Additional file 5 — The hairpin structure of putative TalnpmRNA19. The figure shows the secondary structure of putative wheat long npcRNA TahlnRNA19 by using the Vienna RNA package RNAfold web interface program, the perfect hairpin structure indicates that it might give rise to miRNA. [file 1471-2229-11-61-S5.PDF]

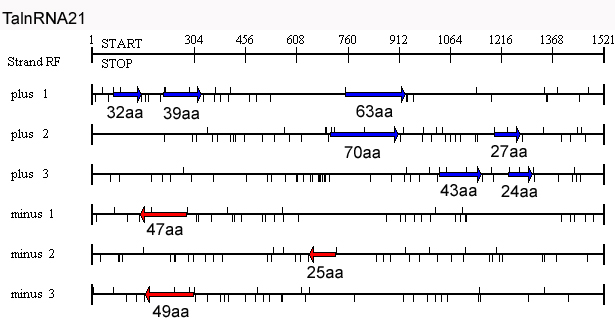

Supplement: Additional file 8 — The short possible ORFs in TalnRNA21. The figure displays all the possible ORFs in the full length cDNA of TalnRNA21, and none of them are longer than 80aa. [file 1471-2229-11-61-S8.JPEG]

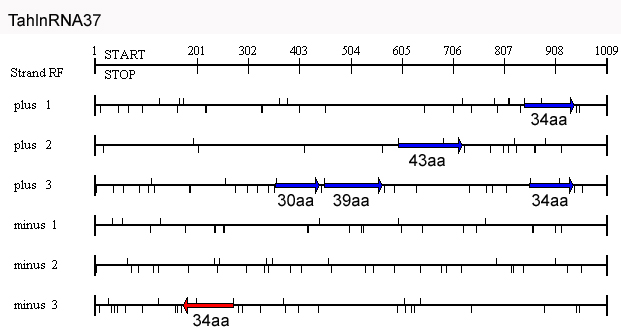

Supplement: Additional file 9 — The short possible ORFs in TahlnRNA37. The figure displays all the possible ORFs in the full length cDNA of TalnRNA37, and none of them are longer than 80aa. [file 1471-2229-11-61-S9.JPEG]

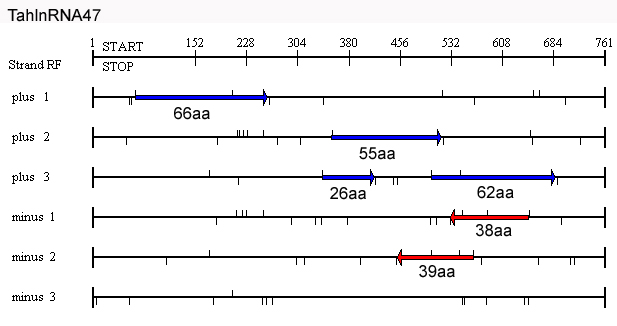

Supplement: Additional file 10 — The short possible ORFs in TahlnRNA47. The figure displays all the possible ORFs in the full length cDNA of TalnRNA47, and none of them are longer than 80aa. [file 1471-2229-11-61-S10.JPEG]
