# Supplementary material for: Identification and characterization of wheat long non-protein coding RNAs responsive to powdery mildew infection and heat stress by using microarray analysis and SBS sequencing
Source: BMC Plant Biol. 2011 Apr 7;11:61. doi: 10.1186/1471-2229-11-61 (PMC3079642; doi:10.1186/1471-2229-11-61)
Supplement: Additional file 6 — Categories of siRNAs corresponding to SRP1 and SRP3 7S RNA variants and sequences of SRP1 and SRP2 corresponding siRNAs. (a) The siRNAs corresponding to SRP1 and SRP3 7S RNA variants are categorized to 5 groups according to their locations, most members of group I, II, and III match both TalnRNA9 and TalnRNA12, and other two (group IV group V) are specific for TalnRNA9. (b) The table includes sequences of of SRP1 and SRP3 corresponding siRNAs [file 1471-2229-11-61-S6.DOC]

**Additional data file 6**

**(a)**: Categories of siRNAs corresponding to SRP1 and SRP3 7S RNA variants

|  | **SRP1 and SRP3** | **SRP1 specific** | **SRP3 specific** |
| --- | --- | --- | --- |
| **GroupⅠ** | **6** | **-** | **2** |
| **GroupⅡ** | **8** | **2** | **-** |
| **GroupⅢ** | **6** | **-** | **-** |
| **GroupⅣ** | **-** | **2** | **-** |
| **GroupⅤ** | **-** | **5** | **-** |

| **GroupⅠ** |  | **GroupⅢ** |  |
| --- | --- | --- | --- |
| Ta2448846 | CTTCCAACGGTGGAAGGATAACGG | Ta2726875 | GGCTTCACAGAGCAGCGACAACTG |
| Ta2296616 | CCCGCTTCCAACGGTGGAAGGATA | Ta2967846 | TCCTTCCACCGTTGGAAGCGGGCA |
| Ta2633187 | GCCCGCTTCCAACGGTGGAAGGAT | Ta3052191 | TGGCTTCACAGAGCAGCGACAACT |
| Ta2053016 | ATCCTTCCACCGTTGGAAGCGGGC | Ta3039140 | TGGAAGCGGGCAGTTGTCGCTGCT |
| Ta2315018 | CCTTCCACCGTTGGAAGCGGGCAG | Ta1433561 | AAGCGGGCAGTTGTCGCTGCTCTG |
| Ta2448878 | CTTCCACCGTTGGAAGCGGGCAGT | Ta1825885 | AGCGGGCAGTTGTCGCTGCTCTGT |
| Ta2960700 | TCCAACGGTGGAAGGATAACGGGC |  |  |
| Ta3106718 | TTCCAACGGTGGAAGGATAACGGG | **GroupⅣ** |  |
| **GroupⅡ** |  | Ta2103276 | ATGCGAAAGCTGGGCCTCACGGTC |
| Ta1888613 | AGGCTGGCTTCACAGAGCAGCGAC | Ta3029400 | TGCCACATTATGGACCGTGAGGCC |
| Ta2560409 | GAGGCTGGCTTCACAGAGCAGCGA |  |  |
| Ta2822642 | GTGAGGCTGGCTTCACAGAGCAGC | **GroupⅤ** |  |
| Ta2395228 | CGTGAGGCTGGCTTCACAGAGCAG | Ta2961410 | TCCACTACGCAACTTGGAACGGGC |
| Ta2662088 | GCGTGAGGCTGGCTTCACAGAGCA | Ta2289040 | CCACTACGCAACTTGGAACGGGCG |
| Ta1828287 | AGCGTGAGGCTGGCTTCACAGAGC | Ta1663154 | ACTACGCAACTTGGAACGGGCGGG |
| Ta1565348 | ACAGCGTGAGGCTGGCTTCACAGA | Ta2906558 | TACGCAACTTGGAACGGGCGGGCC |
| Ta3137945 | TTGTCGCTGCTCTGTGAAGCCAGC | Ta1622619 | ACGCAACTTGGAACGGGCGGGCCA |
| Ta3071472 | TGTCGCTGCTCTGTGAAGCCAGCC |  |  |
| Ta2811354 | GTCGCTGCTCTGTGAAGCCAGCCT |  |  |

**(b)**: Sequences of SRP1 and SRP3 corresponding siRNAs

The number underlined in group Ⅰand group Ⅱ stands for the specific siRNA for SRP1 or SRP3 7S RNA variants
