# Supplementary material for: Identification and characterization of wheat long non-protein coding RNAs responsive to powdery mildew infection and heat stress by using microarray analysis and SBS sequencing
Source: BMC Plant Biol. 2011 Apr 7;11:61. doi: 10.1186/1471-2229-11-61 (PMC3079642; doi:10.1186/1471-2229-11-61)
Supplement: Additional file 7 — Primer sequences for 5'RACE and real time PCR. The table displays the sequences of primers used for both 5'RACE and real time PCR [file 1471-2229-11-61-S7.DOC]

**Supplemental table 3: Primers for 5'RACE and real time PCR**

| **primers for 5'RACE** | |
| --- | --- |
| TahlnRNA47 -outer | 5'-CTGCTGCTATACGGTAGGCTCAGTGAGC-3' |
| TahlnRNA47 -nested | 5'-CTTGAAGTAGAGGCATGGAGCACCTGC-3' |
| TapmlnRNA26-outer | 5'-GCCTGCAGATATGGTACCATGAACTGC-3' |
| TapmlnRNA26-nested | 5'-CTCATTTGTTCACCACATAGAAGCAGGT-3' |
| TalnRNA21-outer | 5'-ATGAAGAGTAGGTCACTAGGGAAGCAAC-3' |
| TalnRNA21-nested | 5'-CTGCGAAGCTTAGTTCTAGAACCATCAC-3' |
| TahlnRNA37-outer | 5’-GTCCACTGTATTCGTCCTCCTGATCCT-3’ |
| TahlnRNA37-nested | 5’-AAGCTAGCAGAAGAAGTTTCTGACTTGC-3’ |
| **primers for quantitative real-time PCR** | |
| TapmlnRNA19-L | 5'-GAGTACCACGTAGCTCTAGATAC-3' |
| TapmlnRNA19-R | 5'-CTGATTCGGACACTTGACAACG-3' |
| TalnRNA5-L | 5'-CTCATTCCTGTCCAGAGGACAT-3' |
| TalnRNA5-R | 5'-CACGTAGTGTATCCTAACATGG-3' |
| TalnRNA9-L | 5'-GTGATGTGGACGCTGTTGTAG-3' |
| TalnRNA9-R | 5'-GTGATGGTTCGCTGGAGTAG-3' |
| TapmlnRNA30-L | 5'-CAAGACGGTGCATCAGGTGTG-3' |
| TapmlnRNA30-R | 5'-TAGCATGCCGGCACTGGAT-3' |
| TahlnRNA37-L | 5'-GAGGATCAGGAGGACGAATAC-3' |
| TahlnRNA37-R | 5'-ACTTGTCGCTGAGTCCATTG-3' |
| TalnRNA2-L | 5’-GCAAGTCTGATCGATTCGAAGC-3' |
| TalnRNA2-R | 5’-AGAGATGTGCTGTGCTGCAC-3' |
| TalnRNA9-anti-L | 5’-GTGATGGTTCGCTGGAGTAG-3' |
| TalnRNA9-anti-R | 5’-CCGTTCCAAGTTGCGTAGTG-3' |
| TalnRNA12-anti-L | 5’-GTTGTCGCTGCTCTGTGAAG-3' |
| TalnRNA12-anti-R | 5’-GGTGTGGACGCTGTTGTAG-3' |
